# Supplementary material for: Effects of the abacus-based mental calculation training application “SoroTouch” on cognitive functions: A randomized controlled trial
Source: PLoS One. 2024 Mar 12;19(3):e0299201. doi: 10.1371/journal.pone.0299201 (PMC10931506; doi:10.1371/journal.pone.0299201)
Supplement: S1 Data — (DOCX) [file pone.0299201.s010.docx]

Trial study protocol

Scientific Title：Effectiveness of an Application Software SoroTouch in Middle and advanced age people （randomized controlled trial）

Version 1.4 September 9th, 2022

Principal investigator: Keiji Hashimoto

Affiliation: Department of Rehabilitation Medicine, Showa University School of Medicine　List of abbreviations and definitions of terms.

| SoroTouch | A digital application for training abacus-based mental calculations developed by Digika Co., Ltd. |
| --- | --- |
| CogEvo^®︎^ | A cognitive function test battery and training tool developed by Total Brain Care Co., Ltd. |
| MoCA-J | The Japanese version of the Montreal Cognitive Assessment |
| SF36 | The 36-Item Short-Form Health Survey |
| MCI | Mild cognitive impairment |

**Contents**

**1.** **Scientific title** 1

**2.** **The organization of the clinical research** 1

**3.** **Backgrounds** 3

**4.** **Objectives** 4

**5.** **Details of the clinical research** 4

**6.** **The inclusion and exclusion criteria and discontinuation criteria for the clinical research** 8

**7.** **Medical treatment of the clinical research participants** 9

**8.** **Evaluations of efficacy** 10

**9.** **Evaluations of safety** 10

**10.** **Statistical analysis** 12

**11.** **Access to original data** 13

**12.** **Quality control and quality assurance** 13

**13.** **Ethical concerns** 14

**14.** **Management and storage of records (including data) and samples** 14

**15.** **Payment and compensation** 16

**16.** **Publication of information** 16

**17.** **Duration of the research** 16

**18.** **Explanation to the research participants and consent to the research** 17

**19.** **Conflicts of interest** 18

**20.** **Intellectual property rights** 19

**21.** **Dealing with personal information** 19

**22.** **Compliance with research protocols and changes to research protocols** 19

**23.** **Management of deviation from research protcols** 20

**24.** **Periodic reporting** 20

**25.** **Termination of research** 20

**26.** **Compliance with clinical research laws** 20

**27.** **Contact details** 21

**28.** **References** 21

1. **Scientific title**

　　　Scientific Title：Effectiveness of an Application Software SoroTouch in Middle and advanced age people （randomized controlled trial）

1. **The organization of the clinical research**

The study will be carried out under the following organization.

**[Principal investigator]**

Name: Keiji Hashimot

Affiliation/Position: Department of Rehabilitation Medicine, Showa University School of Medicine, Associate Professor

Address: 2-1-1 Fujigaoka, Aoba-ku, Yokohama, Kanagawa 〒227-8518

Contact: +81-45-974-2221 ext. 5824

**[Research co-investigator]**

Name: Tetsuya Takaoka

Affiliation/Position: Department of Rehabilitation Medicine, Showa University School of Medicine, Assistant Professor

Name: Nobuyuki Kawate

Affiliation/Position: Department of Rehabilitation Medicine, Showa University School of Medicine, Professor

**[Evaluator]（MoCA-J）**

Name: Kaori Takenaka

Affiliation/Position: Kaori Clinic, Physician

Address: 2F Scinex Headquarters Building, 5-3-16 Uehonmachi, Tennoji-ku, Osaka, 〒543-0001

Contact number: +81-66-770-5407

Activities: conducting the Japanese version of the Montreal Cognitive Assessment (MoCA-J).

Supervision: The MoCA-J is conducted in the consultation room, and the test result forms are converted to PDF by Dr Takenaka, shared on a password-controlled Dropbox and sent to the responsible doctor; once converted to PDF, the forms are immediately destroyed. Finally, Mr Furukawa belonging to St Marianna Medical University checks the PDF of the test form and monitors whether the test has been carried out properly.

**[Evaluator]（CogEvo^®︎^、SF36)**

Name: Kazumi Utamaru

Affiliation / Position: General Incorporated Association, Consortium for Dementia Prevention Activities, Representative

Address: Newspaper Printing Headquarters Building, 4F, 5-17 Higashi Takatsu-cho, Tennoji-ku, Osaka 543-0021, Japan

Contact: mail@niyokatsu.com

Activities: implementation of CogEvo ®︎ and SF-36

Supervision: CogEvo ®︎ and SF-36 will be administered on iPad and questionnaires respectively at the General Incorporated Association office. CogEvo assessment results and SF-36 questionnaires will be converted to PDF by Mrs Utamaru, shared on a password protected Dropbox and sent to the responsible doctor; the converted PDF forms will be destroyed immediately. The PDFs are then promptly destroyed. Finally, Mr Furukawa belonging to St Marianna Medical University checks the PDF of the test forms and monitors whether the tests have been carried out properly.

**[Data management]**

**Responsible organization: Research Center for Advanced Science and Technology, The University of Tokyo**

**Contact person: Sayaka Aoki**

**Affiliation: Department of Human Support Engineering**

**Title: Specially Appointed Researcher**

**Address: Room 310, Building 3, 4-6-1 Komaba, Meguro-ku, Tokyo 153-8904**

**Contact: sayaka-aoki@g.ecc.u-tokyo.ac.jp**

**[Monitoring]**

**Responsible institution: Toyoko Hospital, St. Marianna University School of Medicine**

**Contact person: Toshiyuki Furukawa**

**Affiliation: Syncope Centre**

**Title: Director and Associate Professor of the Centre**

**Address: 2-16-1 Sugao, Miyamae-ku, Kawasaki, Kanagawa 216-8511**

**Contact: +81-44-977-8111**

**[Statistical Analysis]**

**Responsible organization: Research Center for Advanced Science and Technology, The University of Tokyo**

**Contact person: Sayaka Aoki**

**Affiliation: Department of Human Support Engineering**

**Title: Specially Appointed Researcher**

**Address: Room 310, Building 3, 4-6-1 Komaba, Meguro-ku, Tokyo 153-8904**

**Contact: sayaka-aoki@g.ecc.u-tokyo.ac.jp**

1. **Backgrounds**

**3.1 Dementia**

Population projections by the Ministry of Internal Affairs and Communications report that the ageing rate may reach 38.4% by 2065, with approximately 1 in 2.6 people aged 65 or older and 1 in 4 people aged 75 or older. The number of elderly people with dementia is also expected to increase accordingly, and it is estimated that in 2025 the number of elderly people aged 65 and over with dementia will be approximately 7 million, or one in five people.^1)^ Measures for the elderly with dementia will continue to be an ongoing challenge in Japan. However, at present, the effectiveness of drugs to treat dementia is extremely limited, and much is expected from non-pharmacological therapies and preventive measures. Under these circumstances, the long-term care insurance service 'short-term intensive rehabilitation for dementia' was introduced in 2006 as rehabilitation for dementia patients, and reports of its effectiveness in improving cognitive functions and peripheral symptoms of dementia are accumulating.^2-5)^ The individualized program of this 'short-term intensive rehabilitation for dementia' is supposed to last at least 20 minutes per session for one person, and it is estimated that it is often carried out for around 20 minutes in real clinical practice. It is necessary to develop effective rehabilitation equipment that can be implemented in about 20 minutes as an individual program for 'short-term intensive rehabilitation for dementia'.

Mild cognitive impairment (MCI) is a concept that describes a condition in which a person presents with mild cognitive impairment that is not normal but not quite dementia and is able to maintain daily life. If the progression from MCI to dementia can be reduced, it may be possible to reduce the prevalence of dementia in the elderly population.

**3.2** A digital application for training abacus-based mental calculations

'SoroTouch' is a digital application for training abacus-based mental calculations developed to help children acquire high calculation skills efficiently in a short period of time. Science-Technology-Engineering-Math (STEM) is an educational model that began in the 2000s in the USA and is a capability that is attracting attention for the development of human resources for the coming era. SoroTouch is an application that aims to acquire the 'time for maths' and 'ability to work through' that will form the basis of STEM in the new era.

**3.3** **Evidence for SoroTouch**

SoroTouch is an iPad application with a unique interface, called 'invisible mode' (where the beads are not displayed), which requires the learner to visualize and manipulate the beads in his/her mind in order to solve the problems. Furthermore, SoroTouch trains fast and accurate manipulation of the beads and has features that facilitate continuous learning. Analysis of learner data showed that 54% of leavers in 2017 and 62% in 2018 had successfully mastered their mental arithmetic skills by the time they left the program. The time spent using SoroTouch for skill acquisition has been validated to fall between 7 and 26 months.^6)^

**3.4 Hypothesis**

Minami et al. have reported on the usefulness of personal computer (PC)-based attention training for patients with higher brain dysfunction in the chronic phase. Twenty-two patients were divided into a PC-based training group and a conventional drill-based attention training group. After a period of two months (eight sessions), the training methods were switched, resulting in a total intervention period of four months. The results showed that the values of the FIM/FAM cognitive items 'Problem Solving', 'Disability Adaptation' and 'Representation', and the behavioral assessment items 'Triggering' and 'Easy Fatigue' improved significantly after the attention training using the PC.^7)^ In the present study, cognitive rehabilitation with the iPad application SoroTouch may improve participants' cognitive functions, and scientific verification through RCTs is desirable.

1. **Objectives**

The present study is an exploratory clinical study. The subjects will be 20 middle-aged and older adults (40-79 years) and the effects on cognitive functions and quality of life (QOL) will be investigated after they perform SoroTouch for 30 minutes a day for 6 months. In the future, it is expected that SoroTouch will prevent dementia in patients with mild cognitive impairment (MCI).

1. **Details of the clinical research**

**5.1　Primary　outcome and secondary outcome**

5.1.1　Primary outcome

Assessment of five cognitive domains by CogEvo®︎: spatial cognition, orientation, memory, attention and executive function.

5.1.2　Secondary outcome

Quality of life assessment using the SF36 questionnaire

Screening for the presence of dementia with the Japanese version of the Montreal Cognitive Assessment (MoCA-J)

**5.2** **Design and outline of the clinical trial**

5.2.1　Design of the clinical trial

1)　Design ：randomized controlled trial

2)　Intervention type: random allocation

3)　Invasive to subjects: no invasive

5.2.2 Clinical research flow/outline

Registration to allocation should be done within 4 weeks. (Assessment allocation period).

Intervention group（n = 10）

**Intervention period （6 months）**

Control group（n = 10）

Enrolment

Informed consent

Random allocation

The principal investigator explains and obtains consent from the study subjects via online tools such as Zoom in order to prevent the spread of COVID-19 infection. The consent forms are converted into PDF format by the staff of the Consortium for Dementia Prevention Activities, and shared on a password-protected Dropbox and sent to the principal investigator. The paper consent forms are then promptly destroyed.

Schedule of the trials

| Evaluation item | **Registration to allocation should be done within 4 weeks. (Assessment allocation period)** | **One month after** | **Two months after** | **Three months after** | **Four months after** | **Five months after** | **Six months after** |
| --- | --- | --- | --- | --- | --- | --- | --- |
| Obtaining consents | ● |  |  |  |  |  |  |
| Investigation of Characteristics | ● |  |  |  |  |  |  |
| **MoCA-J** | ● |  |  |  |  |  | ● |
| SF36 | ● |  |  |  |  |  | ● |
| CogEvo^®︎^ | ● | ● | ● | ● | ● | ● | ● |
| Investigation of adverse events |  | | | | | | |

The participants will conduct the following curriculum in order from J1, as described in the user manual (Ref. 1). The participants will try to complete each stage, which has 30 missions, three aspects in 30 minutes each day.

The learning time is displayed on the SoroTouch recording screen and the learning time and mission completion status are checked by the staff of the Consortium for Dementia Prevention Activities.

If there are participants whose average daily learning time is less than 10 minutes, the data will be analyzed only for the periods when they were able to do more than 10 minutes.

Ref.1 SoroTouch curriculum

**
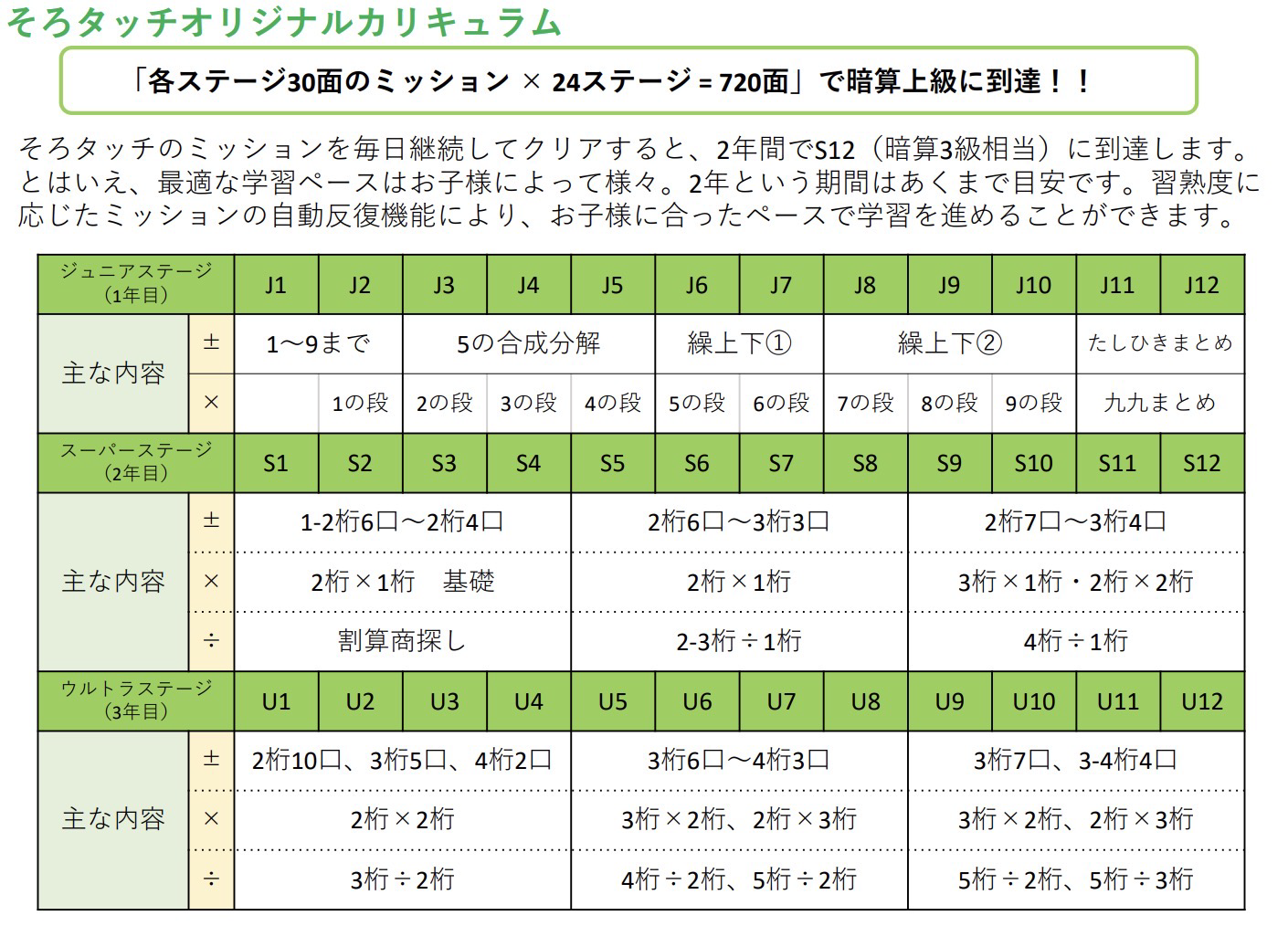
**

**5.3** **Methods of case registration and allocation**

The principal investigator (co-investigator) explains the subject of the study in accordance with the explanatory document and, if consent is obtained, registers and allocates the subject as a research participant. Research participants will be given an identification code for anonymization purposes, which will be used as the identification code for research participants in this study.

The study will be conducted in a randomized comparison to ensure that the allocation does not result in differences in medical background between treatment groups. The participants will be allocated in a 1:1 ratio to the SoroTouch treatment group and the non-treatment group using Muzinwali (https://mujinwari.biz/users/login). The allocation method will be stratified block allocation with CogEvo®︎ (index below and above 70) as allocation factor. After obtaining consent from the study participants, investigators will access Muzinwali to confirm the allocation of the intervention to the study participants concerned. As soon as possible after confirming the allocation, the 'Allocation Instructions/Confirmation Form' is signed and dated. After obtaining consent from the research subject to participate in the research and conducting the allocation, the investigator in charge of the research should ensure that all 'allocation instructions/confirmation forms' are properly kept by the principal investigator.

**5.4** **Medicines-medical devices used in this study**

5.4.1 A digital application for training abacus-based mental calculations ‘SoroTouch’

**5.4.1.1 Functions**

This application has the following functional features.

1. It specializes in the acquisition of imaginative mental calculation skills and is expected to activate the bilateral prefrontal cortex of the cerebrum.
2. The bimanual method is used for effective mental calculation and is expected to activate the bilateral motor areas of the cerebrum.
3. It is an enjoyable application developed by users, which makes it easy to acquire self-learning and maintain motivation.
4. Past studies^6)^ have shown that the system has a proven effect, that over 60% of learners acquire mental arithmetic skills in less than two years.

**5.5** **The expected period of participation of research participants**

From the publication in jRCT to 30 September 2022. It is noted that the trial plan will be published in jRCT after approval by the Showa University School Corporation Clinical Research Review Committee and permission to conduct the research by the head of the implementing medical institution.

**5.6** **Criteria for suspending or discontinuing whole clinical research**

Criteria for suspending or discontinuing the whole clinical trial

If any of the following events occur during the research period, the investigators will discontinue the research on the relevant research participants. When the investigators decide to discontinue or suspend the research, the investigators will take appropriate action to deal with the research participants.

1) When significant information on the quality, safety or efficacy of the equipment used in the study is obtained.

2) When it is deemed difficult to achieve the planned number of cases due to the inability to recruit research participants.

3) When there are instructions from an accredited clinical research review committee to change the research plan, etc., and it is deemed difficult to accept these instructions.

4) When the decision of the accredited clinical research review committee or the administrator of the implementing medical institution instructs to discontinue the research.

5) When a serious or continuous violation of the Clinical Research Act, the Enforcement Regulations or this research protocol occurs.

If the research is to be discontinued, a report will be made to the Authorized Clinical Research Review Committee and the Minister of Health, Labor and Welfare within 10 days of the date of discontinuation. Also, if the research is suspended rather than discontinued, a report will be made to the Authorised Clinical Research Ethics Committee as soon as possible.

**5.7** **How to manage medicines and other products**

Management of the application

This application is to be carried out by the research participants themselves using the iPads loaned by Digika Ltd. The company and the research participant sign a contract based on separate monitoring terms and conditions, and the research participant is responsible for managing the iPad themselves during their participation in the research.

**5.8　Contents directly entered in the case report form and considered source documents**

In this study, the following documents and others are considered source documents (source data).

1) Records of research participant consent and information provided to participants.

2) Information on the presence or absence of diseases, history of diseases, medications taken, etc., as stated in the monitoring application form.

3) Documents or records relating to trials required under the Clinical Research Act in relation to the study.

1. **The inclusion and exclusion criteria and discontinuation criteria for the clinical research**

**6.1** **Target group (diseases)**

　　Middle-aged and elderly adults in the general community

**6.2　 Inclusion criteria**

The subjects must meet all of the following criteria.

1) middle-aged and elderly people who participate in the General Incorporated Association, Consortium for Dementia Prevention Activities.

2) Individuals who are aged between 40 and 79 years when the consent is obtained.

3) Persons who have given their written consent to participate in the study.

［Rationale for setting selection criteria.]

The subjects must be healthy individuals in the age group who are able to continuously carry out SoroTouch.

**6.3　Exclusion criteria**

The subjects must not meet all of the following criteria.

1) Persons with visual or intellectual disabilities that make it difficult to perform SoroTouch.

(2) Persons who have been administered drugs that affect the results of the study to treat dementia or ADHD within six months.

(3) Individuals who are judged to be ineligible by the physician in charge of the study.

[Rationale for setting exclusion criteria.]

(1) Individuals with visual impairment or intellectual disability are difficult to perform the SoroTouch.

(2) Use of drugs that affect cognitive function, as this will affect the results of the study.

3) People who are clearly clinically inappropriate to participate in the study should not participate.

**6.4　 Criteria for discontinuation for each research participant**

If any of the following events occur during the research period, the investigators will discontinue the research on the relevant research participants. When the investigators decide to discontinue or suspend the research, the investigators will take appropriate action to deal with the research participants.

1) If the research participant (or surrogate) asks to discontinue the research.

2) If the research participant discontinues participation in the research for personal reasons (e.g. moving, changing doctor or hospital, busy schedule, inability to follow up, etc.)

3) If the research participants are found to be unsuitable for the subjects after the start of the research

4) If an accidental incident occurs

5) If an adverse event occurs (including exacerbation of the underlying disease, complications or accidental injury) and the investigator decides that the study should be discontinued

6) If it becomes difficult to continue the research due to ill health

7) When there is a serious deviation from the research protocol and it is judged that the research cannot be evaluated

8) When it is found that the research participant is not carrying out the task as instructed by the investigators

9) In other cases where the investigators judge that it is difficult to continue the research and that discontinuation is appropriate.

［Rationale]

This criterion was set in order to ensure the ethical and safe implementation of the research and to ensure the safety of the research participants.

[Methods of action at the time of discontinuation]

After discontinuation, observation will not be continued.

1. **Medical treatment of the clinical research participants**

**7.1　Methods of data collection**

Data obtained at the Kaori Clinic and the Consortium for Dementia Prevention Activities are converted to PDF format by the person in charge, shared on Dropbox with a password set to protect personal data and sent to the principal investigator. Once the PDFs have been converted, the documents are promptly destroyed.

The principal investigator anonymizes the information (including personal data) and creates a correspondence table. The principal investigator stores the correspondence table between individuals and symbols on a computer sealed off from the outside world and on a USB memory stick (mobile media) in the Rehabilitation Department of Showa University Fujigaoka Rehabilitation Hospital. The anonymized Excel file will then be shared on a password-protected cloud with Sayaka Aoki in the Research Center for Advanced Science and Technology, The University of Tokyo, who will do random allocation and statistical analysis.

1. **Evaluations of efficacy**

**8.1　Primary outcome**

Primary outcome

Assessment of five cognitive domains by CogEvo®︎: spatial cognition, orientation, memory, attention and executive function.

Secondary outcome

1. Quality of life assessment using the SF36 questionnaire
2. Screening for the presence of dementia with the Japanese version of the Montreal Cognitive Assessment (MoCA-J)

**8.2　 Evaluation of Efficacy**

8.2.1.　Primary outcome

The intervention is deemed effective if there is a statistically significant difference in changes of CogEvo®︎ scores between the intervention and control groups.

8.2.2.　Secondary outcome

The intervention is deemed effective if there is a statistically significant difference in changes of SF-36 or MoCA-J scores between the intervention and control groups.

**8.3** **Method and timing of analysis of endpoints**

Primary outcome

After entry and initial assessment of study participants, eligible data will be analysed every month for a total of six months.

Secondary outcome

Data will be collected at the end of the evaluation period and MoCA-J and SF36 scores analysed between intervention and control groups.

1. **Evaluations of safety**

**9.1** **Safety assessment measures**

The principal (Co-) investigators evaluate the diseases and defects identified during the period described in '5.5 The expected period of participation of research participants'.

**9.2** **Methods and timing of evaluation, recording and analysis of safety measures**

Investigators will document in the medical record the time of onset and outcome of the disease or defect, severity, outcome, causal relationship to the study and the course of the disease or defect. The severity will be judged according to the following criteria, and the severity will be judged as 'serious' or 'non-serious' based on the criteria in section '9.4 Procedures for collecting, recording and reporting information on diseases and other conditions'.

[Criteria for determining severity]

1) Mild: No treatment is required and the patient recovers.

2) Moderate: Cases in which treatment is required to continue wearing the test device. Cases requiring additional treatment/procedure for post-operative diseases or defects.

3) Severe: Cases in which the continuation of the wearing of the test device is difficult.

**9.3** **Method and timing of analysis on safety measures**

Detailed in section '10. Statistical analyses.

**9.4** **Procedures for collecting, recording and reporting information on diseases and other conditions**

When co-investigators obtain information on the following matters, they report it to the administrator of the implementing medical institution and the principal investigator within the respective timelines stipulated. The principal investigator reports the matter to the accredited clinical research review committee and promptly informs the other research assistants. Then, the other co-investigators will promptly report the matter to the administrator of the implementing medical institution. In addition, when the principal investigator obtains information on the matters in ① and ② (a), he will also report it to the Minister of Health, Labor and Welfare within the stipulated period of time.

①Predictable matters are those described in the study device summary, the study protocol or the consent document. The following diseases etc., the occurrence of which is suspected to be due to this study and which cannot be predicted.

[Reporting period] 7 days.

(a) Death.

(b) Diseases, etc. that may lead to death.

② The following matters in cases where this research is conducted.

[Reporting deadline] 15 days

(a) The following diseases, etc., which are suspected to be caused by this research (excluding those falling under 9.4 ①)

(1) Death

(2) Diseases, etc. that may lead to death

(b) The following diseases, etc., which are suspected to be caused by this research and cannot be predicted (excluding those listed in 9.4 ①)

(1) Diseases requiring hospitalisation or prolonged hospitalisation for treatment, etc.

(2) Disability

(3) Diseases, etc. that may lead to disability

(4) Illnesses, etc. that are serious enough to be related to illnesses (1) to (3) and death or illnesses that are likely to lead to death

(5) Congenital diseases or abnormalities in later generations

③ Diseases suspected to be caused by this research (excluding those listed in ① and ②).

[Reporting deadline] When periodic reports are made to the Authorized Clinical Research Review Committee.

When making reports on ① to ③ above, information should be provided to the company manufacturing the device at the same time.

**9.5** **The observation period for participants in the study after the incidence of disease, etc.**

Research subjects after an outbreak of disease, etc., are followed up until the disease, etc., disappears or until the principal investigator (co-)investigator decides that it is no longer necessary.

1. **Statistical analysis**

If the statistical analysis plan is changed from that established within the protocol, the research protocol should be revised.

**10.1** **Subject of analysis**

10.1.1　 Subjects for efficacy analysis

All randomized subjects, excluding those with no intervention at all and those with no post-intervention assessments at all.

**10.2** **A target number of subjects and rationale for setting the number**

The target number of subjects: 20

［Rationale] Based on the results of the present study on cognitive function and quality of life, a larger-scale validation study is planned in the future. Therefore, this study is an exploratory study, and the number of patients was set at 20 which is the maximum number of patients that can be conducted at the relevant institution. If the intervention in this study has an effect size of 1.4 on the CogEvo change, the probability of error for Type 1 and Type 2 can be assessed as 0.05 and 0.2 respectively with 20 analysis subjects.

**10.3** **Dealing with subjects**

After all subjects have been assessed and prior to analysis, the principal investigator, data management officer and statistical analysis officer will review and record all subjects enrolled in the study, if necessary.

**10.4　Data handling**

If any doubts arise regarding the handling of data during data compilation and analysis, the principal investigator and the statistical analyst will discuss and decide on the handling of the data. Missing values will not be supplemented in principle.

**10.5** **Statistical analysis items and analysis plan**

**10.5.1** **Analysis of study subject background**

Summary statistics for background factors are calculated for the subject population of the efficacy analysis. Specifically, the mean, standard deviation, minimum, median and maximum values are calculated for continuous data, and the number and percentage of persons at each level for classified data.

**10.5.2** **Analysis of primary outcomes**

Group differences in change from baseline on the five cognitive domains (spatial cognition, orientation, memory, attention and executive function) scores of the CogEvo ®︎ will be assessed by analysis of covariance. The significance level is set at 0.05. Missing values are complemented by the last observed value.

**10.5.3** **Analysis of secondary outcomes**

The MoCA-J and SF36 scores will be compared between the two groups before and at 6 months after the start of Soroouch by an unpaired t-test.

**10.6** **Time point of analysis**

For the secondary outcome measures, MoCA-J and SF36 scores, data will be collected during the evaluation period and 6 months after the start of SoroTouch. For the primary outcome, CogEvo®︎, data will be collected during the evaluation period and every month thereafter until 6 months after the start of SoroTouch, and statistical analysis will be performed after the end of the observation period. If there are subjects whose average daily learning time is less than 10 minutes, in principle, the data will be analysed only for the period when they were able to perform the study for more than 10 minutes a day. However, all evaluated data should be used in the analysis. If necessary, subgroup analyses will be conducted according to medical history, diseases being suffered from, medications taken during the intervention period, etc.

The statistical analyst will compile a primary endpoint report and submit it to the principal investigator.

1. **Access to original data**

The principal investigator and the administrator of the implementing medical institution will accept monitoring and investigations by the accredited clinical research review committee and regulatory authorities on this research, and will ensure that all source documents and other materials related to this research are made available for direct inspection at that time.

1. **Quality control and quality assurance**

**12.1　Monitoring**

The principal investigator monitors the research for quality control purposes. He will also prepare a procedure for this purpose and designate a monitor to be in charge of monitoring. The monitor will follow the Monitoring Protocol, which will be prepared separately, to ensure that the study is conducted in compliance with the latest research protocol and regulatory requirements throughout the study period. In addition, the monitor must not divulge personal patient information obtained during monitoring.

**12.2** **Audit**

No audits are carried out.

1. **Ethical concerns**

**13.1** **Benefits arising, anticipated disadvantages and burdens**

The following are possible benefits, disadvantages and burdens arising from participation in this study.

**13.1.1** **Anticipated benefits**

Participants may improve their cognitive functions, such as memory and attention, as a result of performing SoroTouch for a period of six months.

**13.1.2　Expected disadvantages and burdens and measures to minimise them**

Participants in the intervention group may experience psychological strain from performing the SoroTouch for up to 30 minutes a day.

Participants in the control group may also experience time constraints and physical strain from cooperating with the examinations.

The staff of the Consortium for Dementia Prevention and Action will provide support such as talking and encouragement to reduce this burden.

1. **Management and storage of records (including data) and samples**

**14.1** **Preservation of records**

1．The following records should be kept in this study.

1) Information identifying the research participants.

2) Information relating to medical treatment and examination of research participants

3) Information relating to participation in this research

4) Necessary information for conducting this research.

2．The principal investigator must preserve records relating to this research for five years from the date on which this research is completed, with the following documents.

1) Research protocol, documents relating to explanation and consent for research participants, summary report, and other documents or copies of such documents prepared by the principal investigator based on the Clinical Research Act and its implementing regulations.

2) Documents received from the accredited clinical research review committee regarding review opinion

3) Documents related to monitoring

4) Original documents, etc.

5) Contracts or copies regarding the implementation of this research

6) Documents and records prepared or received that outline the medical devices, etc. to be used in this research

7) Documents necessary for conducting this research.

3.　 If the principal investigator revises the record as specified in paragraph 1, he will record the name of the reviser and the date the revision was made, and retain it with the revised record.

**14.2** **Methods of storage and disposal of records**

During the research period, paper records excluding medical records should be stored appropriately in a lockable storage facility under the control and responsibility of the principal investigator. Electronic media records are stored on a computer requiring a password for start-up and on a back-up hard disk, and are stored appropriately under the management responsibility of the principal investigator.

The personal data manager stores the individual case report forms and anonymized clinical data received during the research period appropriately in a lockable storage unit etc.

After the research is completed, the records described in '14.1 Preservation of records' should be recorded on non-rewritable electromagnetic storage media such as CD/DVD or stored in paper form, and stored appropriately for five years in a lockable storage facility under the control and responsibility of the principal investigator. If the principal investigator no longer belongs to the implementing medical institution before the storage period has expired, a person is appointed from persons belonging to the relevant implementing medical institution to store the records. The medical records should continue to be stored in accordance with the rules of the implementing medical institution.

The storage period of the records is five years, and every five years thereafter, the principal investigator discusses the necessity of the extension of the storage period and makes a decision on the extension of the storage period. When the storage period for records ends, the records and data relating to conducting this research will be deleted. At that time, data in the computer should be deleted, and paper materials should be shredded or otherwise destroyed appropriately so that the data and information cannot be recovered. The procedures for storage and destruction of records at each medical institution follows the regulations of the respective medical institution.

During the storage period, the data obtained in this study may still be used for the development of the research equipment. Data obtained in this study should not be used for any purpose except for development of the research equipment.

**14.3** **Storage of information**

During the research period, paper records excluding medical records should be stored appropriately in a lockable storage facility under the control and responsibility of the principal investigator. Electronic media records are stored on a computer requiring a password for start-up and on a back-up hard disk, and are stored appropriately under the management responsibility of the principal investigator.

After the research is completed, the records described in '14.1 Preservation of records' should be recorded on non-rewritable electromagnetic storage media such as CD/DVD or stored in paper form, and stored appropriately for five years in a lockable storage facility under the control and responsibility of the principal investigator. When the storage period for records ends, the records and data relating to conducting this research will be deleted. At that time, data in the computer should be deleted, and paper materials should be shredded or otherwise destroyed appropriately so that the data and information cannot be recovered.

1. **Payment and compensation**

**15.1** **Payment of money (cost-sharing by research participants)**

5,000 per month and a total of 30,000 yen over six months will be paid to all participants in order to reduce the burden on research participants. In addition, the participants will not incur any cost burden in relation to this study, as Digika Ltd. will pay for the consultation fees at the time of MoCA-J implementation and the iPads used in the study.

**15.2** **Compensation**

15.2.1　 Clinical research insurance (indemnity insurance)

The probability of health problems occurring to research participants due to this study is considered to be low. Therefore, it is not considered necessary to subscribe to clinical research insurance and no insurance will be taken out.

**15.2.2** **Compensation for health damage**

Although the probability of health damage occurring to research participants due to this research is considered to be low, the principal ( co-) investigators and the implementing medical institution will provide the best possible medical care and cover medical expenses to ensure that research participants receive appropriate treatment and other necessary measures if serious health damage occurs to research participants as a result of the implementation of this research.

**15.2.3** **Liability insurance coverage**

Liability arising from the general work and medical treatment performed in this clinical research will be covered by medical practitioners' liability insurance. The probability of health damage to research participants arising from this clinical research is considered sufficiently low, therefore no other clinical research insurance for indemnity will be taken out.

1. **Publication of information**

Before conducting this research, information that the World Health Organization requires to be made public in order to conduct clinical research and other information that contributes to ensuring transparency in the process of clinical research and the public's choice to participate in clinical research are recorded in jRCT, a database maintained by the Ministry of Health, Labour and Welfare (https://jrct.niph.go.jp/). The information is recorded and made publicly available. The research will be started after the publication of the 'jRCT', and the information will be updated as appropriate according to changes in the research protocol and the progress of the research. If a primary outcome report or a summary report is prepared, a summary of these will also be made public.

1. **Duration of the research**

・Data collection period: From the date of publication of the study data by the Ministry of Health, Labor to 31 July 2022. (The case registration period is until 30 September 2021).

・Period of study implementation: From the date of publication of the study data by the Ministry of Health, Labor and Welfare to 30 September 2022.

1. **Explanation to the research participants and consent to the research**

The principal investigator or co-investigators give the explanation and consent documents approved by the accredited clinical research review committee to the potential research participant and provide sufficient explanation in writing and orally. The participant fully understands the content of the explanatory document and freely and voluntarily gives written consent. When explaining, the principal investigator and co-investigators ensure that they do not force or unduly influence the research participant to give consent.

The consent document should be signed and dated by each person, stating that the principal investigator and co-investigators have explained and that the research participant has consented to it. The original consent document should be stored appropriately in accordance with the rules of the respective medical institution. A copy of the consent document should also be given to the research participant. Operational details regarding the consent document should be in accordance with the rules of the respective medical institution.

The explanatory document should be carefully worded so that it is easily understood by the participants and should include the following explanatory information.

1) The name of the study, the approval of the administrator of the implementing medical institution for the implementation of the study and the submission of the implementation plan to the Minister of Health, Labour and Welfare.

2) The name of the implementing medical institution and the name and title of the principal investigator (including the name and title of the principal investigator, the name of other implementing medical institutions and the name and title of the principal investigator of those institutions).

3) Reason for being selected as a subject of this study

4) Anticipated benefits and disadvantages of this research

5) Rejection of participation in this research may be freely made.

6) The withdrawal of consent

7) That participants who refuse to participate in this study or withdraw their consent will not be treated unfavorably.

8) Methods of disclosing information on this study.

9) The availability and methods of access to the research protocol and other materials related to this study upon request of the participant.

10) The protection of the personal information of the subjects of this research.

11) Methods of storage and disposal of samples and other materials

12) Matters related to conflict of interest management

13) Matters relating to the response to complaints and enquiries

14) Matters relating to the costs of conducting this research

15） Existence and details of other treatment methods, and comparison of the anticipated benefits and disadvantages of other treatment methods

16) Matters relating to compensation and provision of medical care for damage to health caused by this research

17) Matters to be reviewed by the accredited clinical research review committee, which is responsible for reviewing and giving an opinion on this research, and other matters related to the accredited clinical research review committee involved in this clinical research.

18) Other matters necessary for conducting this clinical research.

When the principal investigator and co-investigators obtain information on efficacy, safety, etc. that affects the consent of research participants, or when the research protocol or other documents are changed that affects the consent of research participants, they will promptly provide the information to the research participants and confirm their willingness to continue participating in this research, which will be recorded in their medical records. The principal investigator will also revise the explanation and consent documents based on the information and obtain approval from the authorized clinical research review committee. After the revision of the explanatory and consent documents has been approved, the principal investigator and co-investigators will promptly obtain the research participant's re-consent in writing.

1. **Conflicts of interest**

**19.1** **Conflicts of interest in research funding and research organisation**

This research is funded by Digika Co., Ltd. and is conducted by Showa University and Digika Co., Ltd. under a subcontract agreement.

The apps used in this research are sold by Digika Co., Ltd. In order to ensure the reliability of the clinical research, Digika Co., Ltd. will not be involved in the selection of subjects for the study or in the acquisition and analysis of data.

Digika Co., Ltd. will pay the fees for clinic visits when conducting MoCA-J and lend iPads to be used in the study free of charge.

Conflicts of interest regarding the research between the researcher and the company are disclosed, and the conflict of interest is reviewed by the Showa University School Corporation Clinical Research Review Committee.

**19.2** **Conflicts of interest of researchers**

The principal investigator prepares a conflict of interests management standard and a report on relevant companies, etc. All principal investigators and co-investigators will be surveyed for conflicts of interest with related companies prior to the start of the study and asked to complete a conflict of interest self-report. The principal investigators will then prepare a conflict of interests management plan based on this. These materials are submitted to the Showa University School Corporation Clinical Research Review Committee for review and approval.

The principal investigator will continuously check with the researcher whether any new 'conflicts of interest' arise in the planning, implementation and reporting of this research that could affect the results of the research and the interpretation of the results.

1. **Intellectual property rights**

The results, data and intellectual property rights obtained from this research will be shared by Digika Co., Ltd. and the principal investigator.

1. **Dealing with personal information**

**21.1** **Protection of personal data**

Careful consideration will be given to the protection of the personal data of research participants when dealing with the data and consent documents of this study. Names, initials, patient IDs at the implementing medical institution, etc. will be treated as personal information and will not be allowed to leave the implementing medical institution under any circumstances. In order to enable the identification of research participants when necessary, the principal investigator will prepare a list of identification codes and their correspondence to research participants, and keep it in strict confidence in accordance with the rules of each implementing medical institution.

In addition, when publishing the results of this study, information that could identify research participants should not be included. Care should also be taken to protect personal data when the data of research participants obtained in this study is used for secondary use in ancillary research conducted in the future.

**21.2　Secondary use of data**

Data obtained in this study may be used secondarily in new studies with the approval of an accredited clinical research review committee or ethics review committee, etc. If the data are stored beyond the period described in 14.2, the personal data of the research participants will be protected as described above. In such cases, the research will be conducted in compliance with the regulations for the purpose of such use and, if necessary, new consent will be obtained or opted out, etc., and the research participant will be provided with the opportunity to refuse secondary use.

1. **Compliance with research protocols and changes to research protocols**

**22.1** **Compliance with the research protocol**

The principal investigators and co-investigators will conduct this research in compliance with the research protocol, as long as the safety and human rights of the research participants are not compromised.。

**22.2** **Changes to the research protocol**

The principal investigator will revise the research protocol if it is deemed necessary to do so. If the research protocol is to be revised, the date on which the revised research protocol will come into effect will be specified, and approval will be obtained from an accredited clinical research review committee. After that effective date, the research will be conducted based on the revised research protocol.

1. **Management of deviation from research protocols**

1) When the principal investigator becomes aware that the study is not in conformity with the Clinical Research Act, the implementing regulations or the research protocol (non-conformity), he will promptly report it to the administrator of the implementing medical institution and inform the principal investigator. In addition, when co-investigators become aware of a non-conformity, he/she will promptly report it to the principal investigator.

2) If a specifically serious non-conformity is found, the principal investigator will promptly report it to the accredited clinical research review committee. If there are other collaborating research organizations, will promptly provide information on it.

1. **Periodic reporting**

Every year from the date of submission of the implementation plan to the Minister of Health, Labour and Welfare, the principal investigator will report on the following issues regarding the conducting of this research to the administrator of the implementing medical institution and the accredited clinical research review committee. He will also report the same to the Minister of Health, Labor and Welfare after hearing the opinions of the Authorized Clinical Research Review Committee.

1) The number of research participants.

2) Occurrence of illnesses, defects, etc. caused by this research and their subsequent progress.

3) Status of occurrence of non-compliance related to this research and subsequent actions taken.

4) Assessment of the safety and scientific relevance of this research.

5) Management of conflicts of interest in relation to this research.

1. **Termination of research**

Within one year after the end of the data collection period of this study, the principal investigator prepares a primary endpoint report, a summary report and its summary. After obtaining the opinion of the accredited clinical research review committee, they will promptly submit them to the administrator of the medical institution where the study was conducted, and publish them on jRCT within one month from the date on which the accredited clinical research review committee has given its opinion.

After submitting the primary endpoint report, the summary report and its summary, the principal investigator will promptly submit the summary of the summary report together with the research plan and statistical analysis plan to the Minister of Health, Labor and Welfare.

1. **Compliance with clinical research laws**

This research will be conducted in compliance with the latest version of the Declaration of Helsinki, the Clinical Research Act, the Enforcement Regulations for the Clinical Research Act (Ministry of Health, Labour and Welfare Ordinance) and various related notifications.

1. **Contact details**

**27.1 Research enquiries**

・Department of Rehabilitation Medicine, Showa University Fujigaoka Rehabilitation Hospital

(2-1-1 Fujigaoka, Aoba-ku, Yokohama, Kanagawa 227-8518, +81-45-974-2221, Keiji Hashimoto (Associate Professor), Office hours 9:00-17:00)

・Kaori Clinic

(5-3-16, Uehonmachi, Tennoji-ku, Osaka, 543-0001, Scinex Headquarters Building 2F, +81-6-6770-5407, Kaori Takenaka (doctor), Office hours 9:30-13:30).

**27.2 Contact point for complaints**

Department of Rehabilitation, Showa University Fujigaoka Rehabilitation Hospital

**27.3 Consultation office of the accreditation review committee that reviewed the application.**

Showa University Educational Corporation Center for Supervision and Research Promotion (Secretariat of the Clinical Research Review Committee of Showa University Educational Corporation)

1-5-8 Hatanodai, Shinagawa-ku, Tokyo 142-8555, Japan

tel: +81-3-3784-8129 (weekdays from 9:00 to 17:00)

E-mail: ura-ec@ofc.showa-u.ac.jp

1. **References**

1) White Paper on Ageing Society, Cabinet Office, 2009 (last viewed 22 Apr 2021). http://www8.cao.go.jp/kourei/whitepaper/w2017/html/gaiyou/gaiyou/index.html

2） K Azuma．Hiyakubutsuryouhou to keihatsukatsudou ninchishoutankishuchu rehabilitation; sono koukano kenshou (in Japanese)．Geriatric Medicine. 2013 ; 51 (1) : 17‒21．

3）Y Nagatomo et al. Ninchishoukoureisha no ninchikinoukaizen ni okeru tankishuchu rehabilitation no kainyukouka (in Japanese)．Journal of the Aichi Society for Physical Therapy. 2011 ; 23 (2) : 51‒55．

4） M Myofuku et al. Ninchisho tannki shuuchu rehabiliration jisshikasann no torkumi ni tsuite ; Heisei 24 nendokaiseimae no matome (in Japanese)．Ishikawaken sagyouryouhougakuzasshi. 2012 ; 20 (1) : 13‒18．

5）S Katoh et al. Kaigoroujinhokenshisetsu ni okeru ninchisho tankishuchu rehabilitation no kouka to sonojizoku (in Japanese). Dementology Updates. 2016 ; 23 (6) : 185‒188．

6) Evaluation of Mental Calculation Educational System with Invisible Abacus, (Last viewed on 13 May 2021.) https://dl.acm.org/doi/abs/10.1145/3395245.3396417" https://dl.acm.org/doi/abs/10.1145/3395245.3396417

7) C Minami et al. Efficacy of generic concentration training in patients with acquired brain injury in the post-acute phase ─comparison between computerized training and workbook exercises, Cognitive Rehabilitation.2013,18(1):19-27.
