# Supplementary material for: Effects of the abacus-based mental calculation training application “SoroTouch” on cognitive functions: A randomized controlled trial
Source: PLoS One. 2024 Mar 12;19(3):e0299201. doi: 10.1371/journal.pone.0299201 (PMC10931506; doi:10.1371/journal.pone.0299201)
Supplement: Attachment — Submitted filename: Plos_one_review.docx [file pone.0299201.s011.docx]

Authors investigated the effects of a home-based computerized cognitive training program on cognitive functions in middle-aged and older adults. The design of the study was randomized controlled trial. In aging society, the topic of the study is current and very important. However, I think that there are some flaws in the study that need to be considered before publishing the study.

Major issues:

1.Throughout the manuscript authors discuss about dementia and preventing dementia. Dementia can be caused by several diseases which all lead to damage in the brain. It is true that several lifestyle factors increase the risk of cognitive decline, however in the case of for example Alzheimer’s disease there is also factors that can not be treated or prevented. Additionally, it is unlikely (unfortunately) that cognitive training without any additional lifestyle intervention would prevent dementia. In the case of this study, in my opinion, it would be better state that computer-based cognitive training may maintain or improve cognitive functions rather than prevent dementia.

2. There is no rationale why authors recruited participants who were at age of 40 to 79. And why they choose cognitively healthy individuals (in study protocol the objectives of the study state that in the future it is expected that SoroTouch will prevent dementia in patients with MCI). Were they at risk of developing cognitive decline?

3. Authors do not provide enough information about participant characteristics (for example, what is the definition of no exercise), training adherence and the way it was monitored and how CogEvo assess the cognitive domains (for example, executive functions is an umbrella term for several sub-domains). Concerning the training adherence how was taken care to ensure that no family member used the exercise program instead of the participant?

4. Sample size in this study was very small and according to Table 1 they were not at risk for cognitive impairment (they were educated, they had normal weight, they exercised, did not use alcohol or smoked and they have almost non chronic diseases that are associated with increased risk of cognitive decline). Therefore, I think that the results of this study can not be generalized outside of this sample.

Minor issues

1. Please use “older adults” instead of elderly

2. Executive functions is an umbrella term for several subdomains and therefore it should be plural.

3. Visuospatial abilities instead of visuospatial cognition

4. Why there is that much missing information in table 2? In this table we unfortunately can see the challenges of small sample size.
