# Supplementary material for: Effects of the abacus-based mental calculation training application “SoroTouch” on cognitive functions: A randomized controlled trial
Source: PLoS One. 2024 Mar 12;19(3):e0299201. doi: 10.1371/journal.pone.0299201 (PMC10931506; doi:10.1371/journal.pone.0299201)
Supplement: Attachment — Submitted filename: Response to Reviewers.docx [file pone.0299201.s012.docx]

Thank you very much for your thorough comments. As you pointed out, because of its design, this study cannot directly test whether the abacus-based mental calculation training application prevents older people from suffering from mild cognitive impairment or dementia. Nevertheless, in Japan, where a super-aging society is emerging, it is crucial to maintain the cognitive function of healthy middle-aged and older individuals as much as possible. Thus, the purpose of this study was restated as investigation of the effect of an abacus-based mental calculation training application on

the cognitive function of healthy middle-aged and elderly individuals.

Reviewer #1: This study uses a home-based abacus training program to train middle aged to elderly individuals, with a 6 month follow up, for improving cognitive functioning. The results show very mild training effect for Sorotech. However, because the statistics are not performed properly, as of now I am not quite convinced that the training effects are there. Comments below.
Response: Thank you for your careful peer review. We have made all possible corrections and would appreciate another peer review.

1. The repeated emphasis of dementia is not necessary because all the participants here are healthy adults, and people with dementia were actually excluded. As such, this is more of a training program for healthy adults. To link this with dementia (or the prevention of it) is too far fetched at the moment.

Response: As you have pointed out, this study was not conducted on patients with dementia or mild cognitive impairment, but on healthy subjects. Therefore, the text has been substantially rewritten as a study that investigated the effect of a cognitive training application on healthy middle-aged and elderly subjects.

See the section of Introduction in p.3-5.

2. This study is characterized as a clinical trial, therefore the trial number from <https://clinicaltrials.gov/> should be provided.

Response: Our study is not registered in <https://clinicaltrials.gov/>, but in the Japan Registry of Clinical Trials (JRCT). The trial ID can be obtained in their website, “https://jrct.niph.go.jp/en-latest-detail/jRCTs032210356.” The information was also added in the last paragraph in the Study Design section (p.7).

3. CogEvo is used as the evaluation tool, but how exactly does it test visuospatial cognition, orientation, memory, executive function, and attention? From my experience, most market softwares that claims ot test these cognitive functions usually blend a lot of unnecessary components into tasks, such that (for example) executive function is conflated with attention, or visuospatial cognition confounded with attention…etc. Therefore, I think the current study is testing a novel training program, and testing it with yet another novel program, so the training effect (or lack thereof) becomes difficult to explain because the cognitive/psychological construct behind each training/testing is unclear.

Response: Thank you for your appropriate comment. Like any other assessment tool, CogEvo is unable to measure one specific cognitive ability without being influenced by other cognitive abilities. However, it is also true that a previous study showed that the item scores of CogEvo are significantly associated with the those of the MMSE, which was designed to measure one’s visuospatial abilities, orientation, working memory, and executive function. Therefore, we decided to describe the outcome of the intervention using the item scores of the CogEvo, not the name of the ability each item is supposed to measure, and add our “interpretation” in the Discussion section. We also provided a task description of each item of the CogEvo more in detail and the strengths of the correlation between the item scores of the CogEvo and those of the MMSE found in a previous study in the CogEvo subsection. Moreover, we also added a video clip to visually demonstrate how an examinee is engaged in each task, as supporting information.

4. Finally and most importantly, it seems that the authors have not done ANOVA or multiple comparison correction of any kind in the results. There are too many t-tests going on, which inflates the chances for false positives. The authors should first perform an ANOVA (with training vs. control, time…etc as factors), and if significant, then move on to posthoc t-tests with (preferably) Bonferroni correction or bonferroni holm correction. Only contrasts that survive ANOVA and posthoc multiple comparison correction should be reported in Results section.

Response: Since a mixed-effects model for repeated measures (MMRM) was used to analyze CogEvo, standard ANOVA could not be applied. Therefore, only the primary endpoint, the total CogEvo score at 6 months, was evaluated for statistical significance, while other evaluations including CogEvo at other time points and secondary endpoints were considered exploratory analyses to avoid multiple comparisons. In exploratory analyses, only p-values were presented without stating whether they were statistically significant or not ("significant" or "not significant" was not mentioned in the manuscript). See the section of Study design in P5-6.

Reviewer #2: Major issues:
1. Throughout the manuscript authors discuss about dementia and preventing dementia. Dementia can be caused by several diseases which all lead to damage in the brain. It is true that several lifestyle factors increase the risk of cognitive decline, however in the case of for example Alzheimer’s disease there is also factors that can not be treated or prevented. Additionally, it is unlikely (unfortunately) that cognitive training without any additional lifestyle intervention would prevent dementia. In the case of this study, in my opinion, it would be better state that computer-based cognitive training may maintain or improve cognitive functions rather than prevent dementia.

Response: Thank you for pointing this out. Referring to your opinion, we have substantially reframed the study as one about whether computer-based cognitive training has a potential to maintain or improve cognitive function in healthy individuals, rather than prevention of dementia (see the Introduction, p.3-5).

2. There is no rationale why authors recruited participants who were at age of 40 to 79. And why they choose cognitively healthy individuals (in study protocol the objectives of the study state that in the future it is expected that SoroTouch will prevent dementia in patients with MCI). Were they at risk of developing cognitive decline?

Response: Thank you for your appropriate comments. In this updated version of the manuscript, we now clearly stated the reason that this study selected people aged 40 to 79 years old as its participants. With regard to the cognitive status of the participants, they do not report any specific risk factors of cognitive decline. However, it is no longer problematic, because we restated the purpose of the study as an investigation of whether SoroTouch improves cognitive function in healthy middle-aged people, taking account of the actual study protocol.

See the section of Participants in P7.

3. Authors do not provide enough information about participant characteristics (for example, what is the definition of no exercise), training adherence and the way it was monitored and how CogEvo assess the cognitive domains (for example, executive functions is an umbrella term for several sub-domains). Concerning the training adherence how was taken care to ensure that no family member used the exercise program instead of the participant?

Response: In response to your comment, we added definitions for each item regarding participant characteristics in the legends in Table 1. Regarding the training adherence, because the participants used SoroTouch online, we were able to know the exact time when Sorotouch was used on each day, but not to confirm who actually used the application, like any home-based online activity without a video-recording. Since this fact should be recognized as one of the limitations of this study, we added it in the Study Limitations in P20.

As for the cognitive domains assessed using the CogEvo, we now added description of each task more in detail and information about the relationship between the item scores of the CogEvo and those of the MMSE in the CogEvo section. Finally, to illustrate the procedure of cognitive assessment in the CogEvomore clearly, we added a video in which an examinee uses CogEvo as supporting information (S1-5 Files).

4. Sample size in this study was very small and according to Table 1 they were not at risk for cognitive impairment (they were educated, they had normal weight, they exercised, did not use alcohol or smoked and they have almost non chronic diseases that are associated with increased risk of cognitive decline). Therefore, I think that the results of this study can not be generalized outside of this sample.

Response: As mentioned in the response to your other comment and at the beginning of this letter, this study is now reframed as a study that investigates the effect of Solotouch on healthy middle-aged adults. Nevertheless, it is true that the small sample size reduces the generalizability of this study. Therefore, we added this concern in the Limitation section (see p.20).

Minor issues

1. Please use “older adults” instead of elderly.

2. Executive functions is an umbrella term for several subdomains and therefore it should be plural.

3. Visuospatial abilities instead of visuospatial cognition.

Response: We have rephrased the terms following your suggestions.

4. Why there is that much missing information in table 2? In this table we unfortunately can see the challenges of small sample size.

Response: As stated in Table 2 legend, some items could not be analyzed for the following reasons: 1) too few people changed; 2) the scores were the same for all; and 3) the scores were not a continuous number, e.g., only 0 or 1. In addition, as mentioned above, this was an exploratory study so we added our understanding that larger sample size trials will need to be conducted in the future, in the Discussion.

Reviewer #3:

Thank you for the opportunity to provide a review of Manuscript PONE-D-23-01459 entitled "EEffects of the abacus-based mental calculation training application “SoroTouch” on cognitive function: a randomized controlled trial". My comments relate primarily to the adequacy of the implementation and reporting of epidemiologic and statistical procedures.

The quality of the technical English is appropriate and offered no bar to my evaluation of the manuscript.

\# Major Issues

\## Abstract
The authors need to quantify the results. They must avoid qualitative statements of effect such as one group experiencing "significantly greater improvement" compared to another. They must report the measure of effect and apply a confidence interval.

Response: We have removed the qualitative statements and added confidence intervals in Abstract in P2.

\## Methods
The authors must identify the primary outcome and all secondary outcomes.

Response: Primary and secondary outcomes were identified and described in the Data analysis in P12 as follows; The primary outcome measure was the total scores of the CogEvo at 6 months after the beginning of the intervention. Secondary outcomes included total scores of the CogEvo at other time points, scores on each subcategory of the CogEvo from 1 to 6 months after the beginning of the intervention, and total scores and scores on each subcategory of the MoCA-J.

The authors fail to report any safety or adverse events outcomes. Why is this missing?

Response: As stated in the Results, one participant contracted COVID-19 unrelated to the present study. Except for this incident, no participants reported any adverse events that prevented them from continuing to participate in the study, such as developing dementia or any other diseases. (Please see the second paragraph of the Result in P15as follows; All participants completed all tests and questionnaires that measured the outcomes of the intervention at all time points of data collection, except for one participant in the control group who was unable to visit the office of the general incorporated association to take CogEvo at 3 months after the beginning of the intervention due to infection with coronavirus disease 2019. )

The authors must report their calculations for sample size.

Response: We have reported the following in Study design section in P5-6 about the sample size calculations as follows; Based on the results of the present study on cognitive function and quality of life, a larger-scale validation study is planned in the future. Therefore, this study was planned as an exploratory study. The number of patients was set at 20, the maximum number of patients that could be conducted at the relevant institution. With this sample size, a two-tailed test at a significance level of 0.05 can detect an effect size of 1.4 with a power of 0.8.

The authors must report standard deviations, not standard errors. The use of standard errors is highly inappropriate.

Response: We reported SD in Table 1. Others reported 95% confidence intervals.

\## Results
The authors MUST NOT apply formal statistical testing between the groups in Table 1. This is highly inappropriate because it sets up a statistical tautology. The last column must be deleted, as well as the last sentence of the footnote.

Response: We deleted the last column in Table 1 in P8 as you have suggested.

Figure 3 is a table and should not be presented as a figure.

Response: Figure 3 has been modified as a table.

In Figure 4, the position of the labels for the horizontal axis is variable. In addition, the scale of the vertical axis is highly variable. These two components of the graph must be fixed for all graphs.

Response: As indicated, the position of the labels for the horizontal axis and the scale for the vertical axis have been fixed.
