# Supplementary material for: Effects of the abacus-based mental calculation training application “SoroTouch” on cognitive functions: A randomized controlled trial
Source: PLoS One. 2024 Mar 12;19(3):e0299201. doi: 10.1371/journal.pone.0299201 (PMC10931506; doi:10.1371/journal.pone.0299201)
Supplement: Attachment — Submitted filename: review_revision.docx [file pone.0299201.s013.docx]

I want to thank the authors for the revised manuscript. I feel that they have successfully improved the manuscript. However, I have still some suggestions how to improve the manuscript.

1. Cognitive functions and executive functions should be plural throughout the manuscript.

2. Reference group should be added in tables that investigate differences between the groups

3. I still feel that it is little bit problematic how executive functions are described in the manuscript. Executive functions is an umbrella term for several subdomains including inhibition, set-shifting and working memory updating. Additionally, Trail Making Test assess some but not all subdomains of executive functions.
