# Supplementary material for: Effects of the abacus-based mental calculation training application “SoroTouch” on cognitive functions: A randomized controlled trial
Source: PLoS One. 2024 Mar 12;19(3):e0299201. doi: 10.1371/journal.pone.0299201 (PMC10931506; doi:10.1371/journal.pone.0299201)
Supplement: Attachment — Submitted filename: Response to Reviewers.docx [file pone.0299201.s014.docx]

We are very grateful for your careful review. Here are the responses to your comments.

Reviewer #1: I appreciate the efforts that the authors have made to address my comments. The introduction now looks okay. However, my most important comment about statistics was ignored by the authors. Multiple comparison or family wise error rate protection should be done, otherwise there is the risk of inflated error rate and hence high false positives. Right now the authors simply claims that everything is exploratory analysis, and still go ahead with an asterisk for p<0.05. I do not agree with the use of this framing to evade multiple comparison corrections. I recommend the authors to use Bonferroni correction, or Bonferroni-holm if Bonferroni is too strict.

Response: Following the recommendations of you and the editor, we applied a Bonferroni correction for our hypothesis testing. As a result, the adjusted criterion for statistical significance of the primary outcome was found to be 0.0014, and p-values smaller than this threshold were considered significant. Referring to the new results obtained after the adjustment, we changed the description in the Data analysis and Results section, Table 2, and Figure 3. (See p.12, 14-16, respectively)

Reviewer #2: I want to thank the authors for the revised manuscript. I feel that they have successfully improved the manuscript. However, I have still some suggestions how to improve the manuscript.

1. Cognitive functions and executive functions should be plural throughout the manuscript.

Response: We have replaced the terms following your suggestions.

2. Reference group should be added in tables that investigate differences between the groups

Response: Due to the space limit, we put just “difference in means” between the 2 groups for each variable at each timing in Tables 2 and Table 3, and actual mean scores and confidence intervals for each group in Tables S1 and S2. If you think it is necessary to provide the mean scores and SD of both SoroTouch group and the Control group in Tables 2 and 3, please let us know.

3. I still feel that it is little bit problematic how executive functions are described in the manuscript. Executive functions is an umbrella term for several subdomains including inhibition, set-shifting and working memory updating. Additionally, Trail Making Test assess some but not all subdomains of executive functions.

Response: Thank you very much for giving us a comment that helps us consider the concepts more thoroughly. After consideration, we decided not to use the term, "executive functions," but directly name the subdomains instead when describing the methods and results of our study. However, we remained to use the term, “executive functions” when we refer to previous studies which adopted the term. As for the Trail Making Test, we rewrote its description to clarify what subdomain of executive function it actually measures (See the section, CogEvo, p.10). In a similar way, we modified the description of the subtest, “Follow the Order” and “Route 99.”

In addition to the revisions above, we also made the data we analyzed available to follow the PLOS data policy. The data are available on Dryad, <https://datadryad.org/stash>. The uploaded dataset included almost all the demographic and clinical characteristics of the participants and the CogEvo and MoCA-J scores. We recoded the age so that the data becomes not personally identifiable.
