# Supplementary material for: Effects of the abacus-based mental calculation training application “SoroTouch” on cognitive functions: A randomized controlled trial
Source: PLoS One. 2024 Mar 12;19(3):e0299201. doi: 10.1371/journal.pone.0299201 (PMC10931506; doi:10.1371/journal.pone.0299201)
Supplement: Attachment — Submitted filename: Review.docx [file pone.0299201.s015.docx]

**General Comments**

Thanks for the invitation to review this article. It was a challenge to review the article after 3 other reviewers had previously reviewed it. The article has potential for publication, considering that it investigates the effect of an intervention with home-based computadorized cognitive function using mental calculation on the improvement/maintenance of cognitive function in healthy middle-aged and older people in a country where the number of older people has grown significantly.

**Major comments**

1. As this is a randomized clinical trial, I suggest a more detailed description of both groups at baseline (table 1). Below is an image that may help with this breakdown.

2. In the supplementary material “S2 table 2”, the “baseline scores” and “amount of change” data are exactly the same for the SoroTouch group. It is important to correct these values.

3. I believe that table 3 can be better explored. More information can be added, such as the mean total MOCA score for the control and intervention groups in the pre- and post-intervention period. For exemplo:

4. An alternative to statistical analysis can be the use of Generalized Estimating Equations (GEE). GEE have been repeatedly applied in controlled clinical trials. GEE and Bonferroni post hoc tests can be used for the comparison between moments (pre- and post-training) and groups.

5. Finally, I think it is important to included in the body and footnotes of the table must describe in detail the statistical method and results of the analysis.
