# Supplementary material for: Effects of the abacus-based mental calculation training application “SoroTouch” on cognitive functions: A randomized controlled trial
Source: PLoS One. 2024 Mar 12;19(3):e0299201. doi: 10.1371/journal.pone.0299201 (PMC10931506; doi:10.1371/journal.pone.0299201)
Supplement: Attachment — Submitted filename: Response to Reviewers.docx [file pone.0299201.s016.docx]

We are very grateful for your careful review. Here are the responses to your comments.

Reviewer #4: The authors have made a lot of efforts on refining this paper based on previous suggestions and now it looks very good.

I have no more questions except that could you provide more information about the intervention in this paper (how many questions solved by participants in the intervention group or ultimately which level did they achieve or frequently choose) to ensure that participants are indeed engaged in the intervention process? Because participants finished the intervention at home where they can be easily distracted, the performance on the calculation tasks may also be important indicators for engagement.

Response: We are grateful for your important suggestion. We agree with the suggestion that the participants' performance on the SoroTouch may have influenced changes in their cognitive functions. We added S1 Table” Performance during the intervention for each participant in the SoroTouch group.” The table includes the usage time, number of answers, number of correct answers, number of wrong answers, and final level of achievement.

Reviewer #5: It is an interesting question to explore the effects of the abacus-based mental calculation training on cognitive function in older people. In the title, authors focused on the effects on cognitive function, but the results did not support the aim or the conclusion of the study. They only observed group differences in two tasks "Follow the order" after 2 months and "Route 99" after 6 months, and this may only be due to familiarity with digits/numbers with SoroTouc training, instead of improvement of working memory or attentional ability.

Response: We agree with your opinion. It is possible that participants' number skills improved rather than their working memory, since "Route99" and "Follow the Order" were similar to SoroTouch in their use of numbers. We have added this description to the Discussion. (See p.19.)

The second question is the sample size was two small, only 10 participants in each group.

Response: We also think the small sample size is a limitation of this study. We have revised the relevant part of the Limitation. (See p. 20.)

it is unclear in the Fig.3, missing SD, line marker etc.

Response: Thank you for your appropriate comments. SE error bars added to Fig 3. SE is shown instead of SD because of the comparison of the population means between the two groups.

Reviewer #6: General Comments

Thanks for the invitation to review this article. It was a challenge to review the article after 3 other reviewers had previously reviewed it. The article has potential for publication, considering that it investigates the effect of an intervention with home-based computadorized cognitive function using mental calculation on the improvement/maintenance of cognitive function in healthy middle-aged and older people in a country where the number of older people has grown significantly.

Major comments

1. As this is a randomized clinical trial, I suggest a more detailed description of both groups at baseline (table 1). Below is an image that may help with this breakdown.

Response: We agree with your comments. 'BMI' and 'Education' in Table 1 were listed as continuous variables. (See p. 8.) As reviewer 3 pointed out as inappropriate at the first review, we did not apply statistical tests between the two groups for characteristics.

2. In the supplementary material “S2 table 2”, the “baseline scores” and “amount of change” data are exactly the same for the SoroTouch group. It is important to correct these values.

Response: We appreciate your suggestion. We have corrected that part.

3. I believe that table 3 can be better explored. More information can be added, such as the mean total MOCA score for the control and intervention groups in the pre- and post-intervention period. For exemplo:

Response: We have added the MoCA-J mean scores for both groups pre- and post-intervention in Table 3, following your suggestion. (See p. 16-17.)

4. An alternative to statistical analysis can be the use of Generalized Estimating Equations (GEE). GEE have been repeatedly applied in controlled clinical trials. GEE and Bonferroni post hoc tests can be used for the comparison between moments (pre- and post-training) and groups.

Response: We agree with your comments regarding GEE. However, we planned this trial with a mixed-effect model approach, so we analyzed data by MMRM. Additionally, we don't consider there are any drawbacks to analyzing the data with MMRM.

5. Finally, I think it is important to included in the body and footnotes of the table must describe in detail the statistical method and results of the analysis.

Response: As you indicated, we have described the statistical methods and results of the analysis in detail in the footnotes of the tables.
